# Submucosal hyper-echogenicity on intestinal ultrasound is associated with fat deposition and predicts treatment non-response in patients with ulcerative colitis

**DOI:** 10.1093/ecco-jcc/jjaf158

**Published:** 2025-11-04

**Authors:** Maarten J Pruijt, E Andra Neefjes-Borst, Floris A E De Voogd, Marilyne M Lange, Christoph Teichert, Reimer J Janssen, Geert R D’Haens, Krisztina B Gecse

**Affiliations:** Department of Gastroenterology and Hepatology, Amsterdam UMC, Amsterdam, The Netherlands; Department of Pathology, Amsterdam UMC, Amsterdam, The Netherlands; Department of Gastroenterology and Hepatology, Amsterdam UMC, Amsterdam, The Netherlands; Department of Pathology, Amsterdam UMC, Amsterdam, The Netherlands; Department of Gastroenterology and Hepatology, Amsterdam UMC, Amsterdam, The Netherlands; Department of Gastroenterology and Hepatology, Amsterdam UMC, Amsterdam, The Netherlands; Department of Gastroenterology and Hepatology, Amsterdam UMC, Amsterdam, The Netherlands; Department of Gastroenterology and Hepatology, Amsterdam UMC, Amsterdam, The Netherlands

**Keywords:** intestinal ultrasound, ulcerative colitis, submucosal hyper-echogenicity

## Abstract

**Background and Aims:**

The submucosa is the most responsive bowel wall layer on intestinal ultrasound (IUS) when assessing treatment response in ulcerative colitis (UC). Submucosal thickening with hyper-echogenicity is observed. This study aimed to quantify echogenicity and understand transmural changes in UC.

**Methods:**

In total, 118 patients were studied in two cohorts. Cohort 1 included colectomy patients: 19 UC patients and 52 controls without inflammatory bowel disease. Cohort 2 included 47 UC patients in a prospective cohort starting anti-inflammatory treatment. In Cohort 1, submucosal inflammation, and fat and collagen deposition were scored by two pathologists using a semi-quantitative scale (0–3). For UC patients in Cohort 1, histopathology and IUS of the sigmoid were location matched. Relative submucosal echogenicity (RSE) was assessed, quantified in grayscale values. In Cohort 2, baseline sigmoid RSE was compared between endoscopic responders (≥1 point decrease in endoscopic Mayo score after 8–26 weeks) and non-responders.

**Results:**

In all colectomized UC patients with preserved wall layer stratification (*n* = 12, 63%), submucosal fat (score ≥1) was present; in those with loss of stratification (*n* = 7, 37%), fat was absent (score = 0). RSE was higher when fat was present [95.5 (IQR 86.5–116.9) vs 8.1 (IQR 5.8–23.0) grayscale values, *P* < .001], with no significant differences for inflammation and collagen. In Cohort 2, RSE was higher in non-responders (*n* = 17) compared to responders (137.1 ± 50.9 vs 88.3 ± 49.6 grayscale values, *P* = .003). An RSE of >108 grayscale values predicted non-response [OR: 0.07 (95% CI: 0.01–0.44), *P* = .004].

**Conclusion:**

Submucosal hyper-echogenicity on IUS indicates fat deposition and predicts non-response in UC.

## Graphical abstract

**Graphical Abstract:**
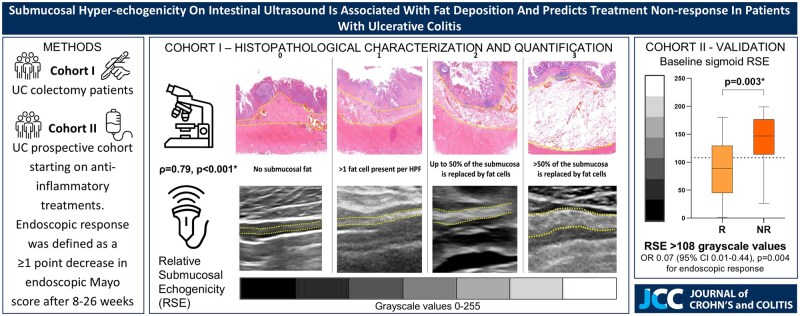

## 1. Introduction

Ulcerative colitis (UC) is traditionally regarded as a chronic inflammatory disease limited to the mucosal layer of the bowel wall. Accordingly, current long-term treatment goals are based on endoscopic mucosal healing.^1^ However, increasing evidence suggests that UC is also characterized by transmural changes.^2^ Submucosal fat and collagen deposition have been described in the resection specimen of UC patients.^3^^,^^4^ Intestinal ultrasound (IUS) is a cross-sectional imaging technique that is frequently used to assess disease activity in patients with UC.^5^ Whereas measurement of total bowel wall thickness (BWT) is the most important ultrasound parameter for evaluating inflammation, IUS also visualizes changes in individual bowel wall layers.^6^^,^^7^ Recent studies found that the submucosal layer is the most thickened in inflammation, and is also most responsive to anti-inflammatory treatment in UC.^6^ However, based on clinical observations, some UC patients exhibit persistent submucosal thickening, which is characterized by hyper-echogenicity.^2^ Here we aimed to (1) characterize the underlying transmural histopathological changes associated with a thickened, hyperechogenic submucosal thickening, (2) quantify submucosal echogenicity, and (3) identify the implications of these structural changes in treatment outcomes.

## 2. Materials and methods

In this study, 118 patients were included across two cohorts. Cohort 1 included UC patients and controls without inflammatory bowel disease (IBD) who all underwent colectomy. Cohort 2 included UC patients in a prospective, validation cohort who were starting on anti-inflammatory treatment.

### 2.1. Cohort 1

#### 2.1.1. UC colectomy patients

We included UC patients who underwent colectomy and had undergone IUS within 3 months prior to surgery. We identified consecutive UC patients from our outpatient clinic or hospital electronic records who had a complete IUS examination before a subtotal colectomy between April 2019 and March 2024. The inclusion criteria were: (1) patients with an established diagnosis of UC, (2) who underwent colectomy, (3) who had an IUS examination within 3 months prior to surgery, (4) with available histopathological slides of the sigmoid colon, and (5) who provided informed consent. The exclusion criteria were: (1) incomplete IUS examination due to poor quality or missing images of the sigmoid, and (2) no histopathological slides available or poor quality of slides. The indications of colectomy were acute severe UC, therapy refractory UC, and/or dysplasia.

#### 2.1.2. Non-inflammatory bowel disease colectomy control patients

To compare histopathological findings in UC with non-IBD patients, we identified control patients who underwent a colectomy or segment resection. We included: (1) control non-IBD patients matched for age and sex with our UC colectomy group, (2) elderly non-IBD patients (60 years and older), and (3) non-IBD patients with a history of diverticulitis. We identified the control patients using the pathology registry system in the Amsterdam University Medical Center (Amsterdam UMC). Exclusion criteria were (1) concomitant auto-immune disease, (2) any other cause of (chronic) inflammation including ischemia (except for the diverticulitis group), and (3) neo-­adjuvant chemotherapy and immunotherapy.

### 2.2. Cohort 2

We validated our findings using a prospective cohort of patients enrolled in a study conducted at the Amsterdam UMC.^6^ This observational cohort included UC patients starting on any anti-inflammatory treatment, receiving at least endoscopy and IUS at baseline and during follow-up (between weeks 8 and 21). Endoscopic response and remission was defined as a  ≥ 1 point decrease in endoscopic Mayo score (EMS) and EMS of 0, respectively, at follow-up.

The Institutional Review Board of the Amsterdam UMC proved this study (EC number 2023.0128). Informed consent was obtained for all patients. In Cohort 2, patients signed informed consent for the prospective study as approved by the Institutional Review Board of the Amsterdam UMC.^6^

### 2.3. Procedures

#### 2.3.1. Intestinal ultrasound

IUS images from the sigmoid colon in all UC patients were reviewed by one ultrasonographer (MP, 3 years of experience) blinded for clinical, biochemical and pathology data using a DICOM-viewer [RadiAnt DICOM Viewer (Software) v.2016]. All images and cine-loops of the sigmoid colon were measured for all IUS parameters as described in Table S1. To quantify submucosal echogenicity, the mean areal grayscale values (a numerical scale of 0–255, with zero corresponding to black) for the submucosa and muscularis propria was measured in a DICOM-viewer. To correct for variations in depth and gain, the relative submucosal echogenicity (RSE) was calculated by subtracting perpendicular measurements in the submucosa and muscularis propria. Each measurement was performed twice within one still image with ≥10 mm between measurements, and RSE was calculated as ((2 × measurement submucosa)/2)) −((2 × measurement muscularis propria)/2)) (Figure S1). In cases of wall layer stratification loss, adjacent segments with preserved layers were used to identify the muscularis propria and submucosa for accurate RSE assessment. All IUS examinations were performed with an Epiq 5G ultrasound scanner (Philips, The Netherlands) using a C5-1 MHz, L12-5 MHz, and/or L18-4 MHz probe. Gain, frequency, and focus settings were optimized per patient. A velocity scale of 5 cm/s was used to measure color Doppler signal (CDS).

A second reader (FdV, 6 years of experience) scored the RSE again in all UC patients of Cohort 1. The results from the first reader (MP) were used for further analysis. The results from the second reader (FdV) were used to assess inter-observer agreement.

#### 2.3.2. Histopathology

Two pathologists (AN and ML with >30 and >2 years of experience in gastrointestinal pathology), blinded for clinical, biochemical, and ultrasonographic outcomes, scored the Nancy score and submucosal inflammatory infiltrate, fat, and collagen in Cohort 1 in a semi-quantitative manner described in Table S2. In UC colectomy patients, histopathological slides of the sigmoid colon were location matched with the IUS images. The results from the first reader (AN) were used for further analysis. The results from the second reader (ML) were used to assess inter-observer agreement.

### 2.4. Statistics

Statistical analysis was performed with SPSS Statistics for Windows, v.28 (IBM Corp., Armonk, NY, USA). All normally distributed data were reported as mean ± SD and non-normally distributed as median and IQR. Mann–Whitney U tests were used to compare continuous non-parametric variables, chi square tests for dichotomous variables, and Wilcoxon rank tests or McNemar tests for paired samples. Relationships were examined using Spearman correlation; correlation coefficients and significance levels were reported. Inter-observer agreement was assessed using Cohen’s kappa statistics, Fleiss’ kappa statistics, or intra-class coefficient (ICC) for dichotomous, ordinal, or continuous data, respectively.^8^ Linear regression analyses were conducted to investigate relationships between parameters; regression coefficients, SEs, and significance levels were reported. Logistic regressions were employed to assess the predictive values of parameters. Odds ratios (ORs) with CIs and significance levels were reported. To adjust for potential confounders, multivariable logistic regressions were performed. Adjusted ORs with CIs and significance levels were reported. Receiver operating characteristic (ROC) analysis was performed to determine cut-offs, the area under the curves (AUC) were reported. A significance level of <0.05 was considered statistically significant.

## 3. Results

In total, 118 patients were included in this study. Cohort 1 included 71 patients: 19 with UC and 52 controls. Cohort 2 included 47 UC patients (Figure S2).

### 3.1. Cohort 1

#### 3.1.1. UC patients undergoing colectomy

Nineteen UC patients undergoing colectomy were included (Table 1). Indications for colectomy were therapy-refractory UC (12/19, 63%), acute severe colitis (6/19, 32%), and dysplasia (1/19, 5%). Median time between IUS and surgery was 11.5 days (range 0–94). Eleven patients (12/19, 63%) failed at least two biologicals and/or small molecules.

**Table 1.** Demographic characteristics of the UC patients included in Cohort 1.

Variable	UC patients (*n* = 19)
**Female, *n* (%)**	8 (42%)
**Age at diagnosis (years), median (range)**	27 (13–40)
**Disease duration (years), median (range)**	8 (0–35)
**Age at colectomy (years), median (IQR)**	39 (26–48)
**Smoking status, *n* (%)**	
** Active smoker**	1 (5%)
** Former smoking**	6 (32%)
** Non-smoker**	12 (63%)
**Montreal classification, *n* (%)**	
** Left-sided colitis (E2)**	3 (16%)
** Pancolitis (E3)**	16 (84%)
**Previous biologicals/small molecules, *n* (%)**	
** None**	6 (32%)
** One**	1 (5%)
** Two**	3 (16%)
** Three or more**	9 (47%)
**Current treatment, *n* (%)**	
** Oral 5-ASA**	10 (53%)
** Systemic corticosteroids**	14 (74%)
** Infliximab monotherapy**	2 (11%)
** Infliximab + immunomodulator**	3 (16%)
** Vedolizumab**	2 (11%)
** Ustekinumab**	1 (5%)
** Tofacitinib**	6 (32%)
** Upadacitinib**	1 (5%)
** Filgotinib**	1 (5%)
** Ozanimod**	1 (5%)
**Number of patients on advanced therapy, *n* (%)**	17 (90%)
**Colectomy indications**	
** Therapy-refractory UC**	12 (63%)
** Acute severe UC**	6 (32%)
** Dysplasia**	1 (5%)
**Time between IUS and colectomy (days), median (range)**	11.5 (0–94)

Abbreviations: UC, ulcerative colitis; IQR, inter-quartile range; 5-ASA, 5-aminosalicylic acid; IUS, intestinal ultrasound.

At IUS, median BWT was 4.5 mm (IQR 4.3–4.8) with a median submucosal thickness of 1.8 mm (IQR 1.5–2.2). Loss of stratification was seen in seven patients (7/19, 37%). At histopathology, submucosal inflammation (category ≥1) was present in 18 patients (18/19, 95%), submucosal fat (category ≥1) in 12 patients (12/19, 63%), and submucosal collagen (category ≥1) in 18 patients (18/19, 95%; Table S3). All patients with loss of stratification had absence of fat deposition (category = 0; 7/7, 100%) but severe mucosal disease (Nancy score of 4; 7/7, 100%; Table S3). All patients with preserved wall layer stratification had presence of submucosal fat, while fewer than half of the patients had a Nancy score of 4 (5/12, 42%; Table S3).

**Figure 1.**
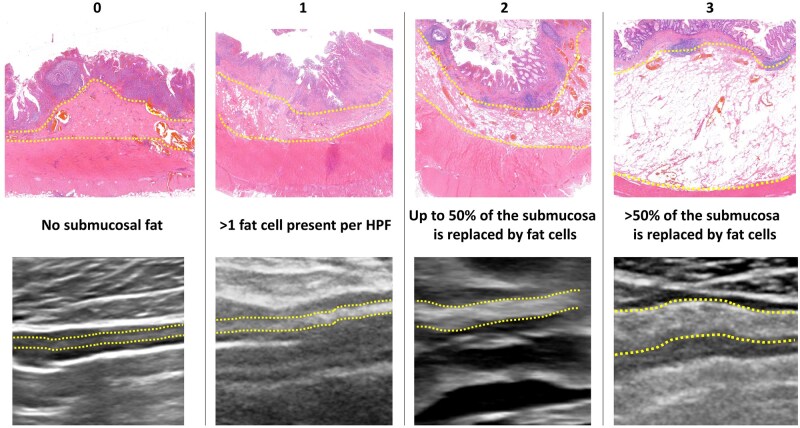
Scale for scoring histopathological presence of submucosal fat and the corresponding IUS images. Yellow dotted lines indicate the submucosa layer. HPF, high-power field; IUS, intestinal ultrasound.

A substantial correlation was found between wall layer stratification and fat deposition [ρ = 0.80 (0.53–0.92), *P* < .001; Figure 1, Figure S3]. For submucosal collagen there was a moderate correlation [ρ = 0.54 (0.02–0.09), *P* = .018] with wall layer stratification, and no correlation was found for submucosal inflammatory infiltrate.

**Figure 2.**
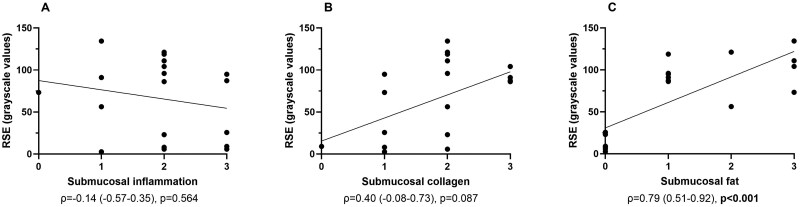
Correlation between RSE and histopathological scoring of inflammation (A), collagen (B), and fat (C) in UC patients in Cohort 1. RSE, relative submucosal echogenicity; UC, ulcerative colitis.

A substantial correlation was found between RSE and fat deposition (ρ = 0.79 (0.51–0.92), *P* < .001], but not for the other histopathological parameters (Figure 2). We observed a substantial correlation with RSE and wall layer stratification [ρ = 0.87 (0.67–0.95), *P* < .001].

Univariable linear regression indicated that both fat deposition on histopathology (B = 30.38, *P* < .001) and collagen (B = 27.48, *P* = .031) in the submucosa were associated with a higher RSE (Table S4). In the multivariable linear regression, only fat deposition remained significantly associated with RSE (B = 31.27, *P* < .001; Table S4).

RSE was significantly higher in patients with fat deposition compared to those without fat deposition [95.5 (IQR 86.5–116.9) vs 8.1 (5.8–23.0) grayscale value, *P* < .001). Submucosal thickness was significantly higher in patients with fat deposition [2.1 mm (IQR 1.8–2.6) vs 1.4 mm (IQR 1.2–1.8); *P* = .010], while BWT was similar between these groups [4.5 mm (4.4–4.7) vs 4.3 mm (4.2–5.3); *P* = .71; Table 2).

**Table 2.** Ultrasonographic and demographic characteristics of patients with submucosal fat deposition versus without fat deposition.

Histopathological presence of submucosal fat			
Variable	Submucosal fat deposition (*n* = 12)	No fat deposition (*n* = 7)	*P*-value
**Female**	4 (33.3)	4 (57.1)	.38
**Age at time of colectomy (years)**	28.0 (24.0–48.5)	43.0 (35.0–44.0)	.26
**Age at diagnosis (years)**	24.0 (18.0–33.0)	32.0 (27.0–38.0)	.10
**Disease years (years)**	5.0 (3.0–13.0)	8.0 (0.0–19.0)	.97
**Failed >1 biological, *n* (%)**	9 (75.0)	3 (42.9)	.33
**BWT, mm, median (IQR)**	4.5 (4.4–4.7)	4.3 (4.2–5.3)	.71
**Submucosal thickness, mm, median (IQR)**	2.1 (1.8–2.6)	1.4 (1.2–1.8)	**.010**
**Submucosa/BWT percentage (%)**	46.2 (42.5–51.9)	29.1 (20.1–41.8)	**.004**
**RSE, grayscale value, median (IQR)**	95.5 (86.5–116.9)	8.1 (5.8–23.0)	**<.001**

Statistically significant values are shown in bold.Abbreviations: BWT, bowel wall thickness; RSE, relative hyperechogenicity of the submucosa.

#### 3.1.2. Non-IBD patients undergoing resection

A total of 52 control patients were included (Table S5). Indications for surgery included cancer (31/52, 59%), diverticulitis (17/52, 33%), and refractory obstipation (4/52, 8%). Patients were divided into three groups: (1) non-IBD patients, age and sex matched with the UC patients (*n* = 18), (2) non-IBD patients 60 years or older (*n* = 17), and (3) non-IBD diverticulitis patients (*n* = 17).

No significant differences were seen in fat and collagen between UC patients and any control groups (Tables S5 and S6). Only submucosal inflammation differed significantly between the groups. While in UC nearly all patients (95%) demonstrated submucosal inflammation, no inflammation was observed in group 1 or 2, and only in two diverticulitis patients in group 3 (Table S6).

In all colectomy patients combined (*n* = 71), males scored significantly higher for the presence of submucosal fat compared to females, with category distributions as follows: 0 (*n* = 8 vs 12), 1 (*n* = 6 vs 12), 2 (*n* = 16 vs 6), and 3 (*n* = 7 vs 4), respectively (*P* = .045; Table S7). No significant differences in fat deposition were seen based on patient age (Table S7).

#### 3.1.3. Inter-observer agreements

We found a substantial inter-observer agreement for RSE [ICC: 0.81 (95% CI 0.56–0.92), *P* < .001; Table S8). Inter-observer agreements of histopathological parameters were moderate for submucosal fat [weighted kappa: 0.50 (95% CI 0.30–0.71), *P* < .001] and fair to moderate for Nancy [weighted kappa: 0.41 (0.05–0.77), *P* = .012], submucosal inflammation [weighted kappa: 0.22 (−0.2 to 0.47), *P* = .031], and collagen [weighted kappa: 0.42 (0.17–0.67), *P* = .005] (Table S8).

### 3.2. Cohort 2

#### 3.2.1. Moderate to severe UC patients starting on anti-inflammatory treatment

A total of 47 patients with moderate to severe UC were included in this prospective validation cohort (Table 3). Nineteen patients previously failed at least two biologicals (41%). Of these, nine patients started on infliximab treatment (9/47, 19%), six on vedolizumab (6/47, 13%), one on ustekinumab (1/47, 2%), and 29 on tofacitinib (29/47, 62%), and two patients on systemic corticosteroids only (2/47, 4%). Of the 47 patients, 45 went through a follow-up endoscopy to evaluate treatment response. In total, 28 patients (28/45, 62%) were endoscopic responders and 13 patients reached endoscopic remission (13/45, 29%).

**Table 3.** Characteristics of the prospective UC cohort.

Demographics UC prospective cohort	
Variable	UC patients (*n* = 47)
**Female, *n* (%)**	25 (53%)
**Age (years), median (IQR)**	37.0 (27-51)
**Age at diagnosis (years), median (IQR)**	29.0 (22–39)
**Disease duration (years), median (range)**	7.0 (3–11)
**BMI, mean (SD)**	23.4 (4.8)
**Montreal classification**	
** Proctitis (E1)**	2 (4%)
** Left-sided colitis (E2)**	23 (49%)
** Pancolitis (E3)**	22 (47%)
**EMS of the sigmoid at baseline^a^**	
** Mayo 0**	2 (4%)
** Mayo 1**	1 (2%)
** Mayo 2**	20 (44%)
** Mayo 3**	22 (49%)
**Previous biologicals/small molecules**	
** None**	18 (38%)
** One**	10 (21%)
** Two**	12 (26%)
** Three**	7 (15%)
**Treatment**	
** Systemic 5-ASA**	18 (38%)
** Systemic corticosteroids**	31 (66%)
** Infliximab monotherapy**	2 (4%)
** Infliximab + immunomodulator**	7 (15%)
** Vedolizumab**	6 (13%)
** Ustekinumab**	1 (2%)
** Tofacitinib**	29 (62%)
**CRP (mg/L), mean ± SD**	26.0 (46.6)
**Fecal calprotectin (µg/g), mean ± SD**	2449.5 (2152.2)
**Endoscopic response^a^ (decrease in EMS of ≥1), *n* (%) **	28 (62%)
**Endoscopic remission^a^ (EMS = 0), *n* (%) **	13 (29%)
**Time between baseline and follow-up endoscopy, weeks (IQR)**	9.6 (8.7–20.4)

Abbreviations: UC, ulcerative colitis; EMS, endoscopic Mayo score; BMI, body mass index; CRP, C-reactive protein; 5-ASA, 5-aminosalicylic acid. Endoscopic response (decrease of at least 1 EMS), endoscopic remission (EMS = 0). ^a^Data available for *n* = 45.

In endoscopic responders, baseline RSE was significantly lower compared to non-responders (88.3 ± 49.6 vs 137.1 ± 50.9 grayscale value, *P* = .003). RSE at baseline was higher in patients who failed more than one biological compared to those who failed one or fewer (Figure S4).

Between RSE and IUS parameters, we demonstrated a weak negative correlation with BWT [ρ = −0.31 (−0.54 to 0.02), *P* = .036] and a moderate negative correlation with loss of stratification [ρ = −0.42 (−0.63 to −0.14), *P* = .004; Table S9]. A moderate negative correlation was observed between RSE and CRP at baseline [ρ = −0.40 (−0.67 to −0.02), *P* = .034]. No correlation was observed between RSE and age, disease duration, and body mass index (BMI) (Table S9).

Univariable logistic regression analysis for predicting endoscopic response in the sigmoid at baseline identified RSE and fecal calprotectin (FCP) as significant predictors of endoscopic response [OR 0.98 (0.97–0.99), *P* = .007 and 1.00 (1.00–1.001), *P* = .049, respectively; Table 4]. The ROC curve analysis for endoscopic response in the sigmoid demonstrated a significant AUROC for RSE [0.763 (0.61–0.92), *P* = .003], while FCP only showed a trend [0.677 (0.51–0.84), *P* = .057; Figure S5]. The optimal cut-off values for predicting endoscopic response were RSE > 108 grayscale value and FCP > 1520 µg/g (Table S9), corresponding to univariable ORs of 0.12 (95% CI 0.027–0.516, *P* = .004) and 3.8 (95% CI 1.011–13.91, *P* = .048), respectively (Table 4). In a multivariable model, RSE > 108 grayscale values and FCP> 1520 µg/g remained significant and demonstrated an OR of 0.072 (95% CI 0.01–0.45, *P* = .004) and 7.18 (95% CI 1.27–40.54, *P* = .026), respectively (Table 4).

**Table 4.** Results of logistic regression analysis for endoscopic response (EMS decrease of ≥1) of biochemical and IUS characteristics at baseline; backward selection.

Logistic regression for endoscopic response (EMS decrease of ≥1 or more)				
Variables at baseline	Univariable analysis OR (95% CI)	*P*-value	Multivariable analysis OR (95% CI)	*P*-value
**RSE (grayscale values)**	0.980 (0.966–0.995)	**.007**	–	–
**RSE >108 (grayscale values)**	0.119 (0.027–0.516)	**.004**	0.072 (0.012–0.442)	**.004**
**BWT (mm)**	1.440 (0.904–2.295)	.125	–	–
**BWT >5.53 (mm)**	4.853 (0.92–25.5)	.062	–	–
**Submucosal thickness (mm)**	1.311 (0.667–2.575)	.432	–	–
**CDS (per category increase)**	2.147 (0.90–5.12)	.085	–	–
**CDS (mLimberg 2 or 3)**	2.564 (0.497–13.220)	.260	–	–
**Loss of stratification**	2.817 (0.734–10.805)	.131	–	–
**Loss of haustration**	4.000 (0.646–24.768)	.136	–	–
**Presence of lymph nodes**	3.300 (0.936–11.631)	.063	–	–
**CRP (mg/L)**	1.035 (0.974–1.101)	.270	–	–
**FCP (µg/g)**	1.000 (1.000–1.001)	**.049**	–	**–**
**FCP >1520 (µg/g)**	3.750 (1.011–13.91)	**.048**	7.18 (1.27–40.54)	**.026**

Statistically significant values are shown in bold.Abbreviations: EMS, endoscopic Mayo score; RSE, relative hyperechogenicity of the submucosa; BWT, bowel wall thickness; CDS, color Doppler signal; CRP, C-reactive protein; FCP, fecal calprotectin.

Male sex, failing more than one biological and having an EMS of 3 was significantly associated with an RSE > 108 grayscale value [OR 4.7 (1.4–16.5), *P* = .014, OR 4.3 (1.2–15.4), *P* = .024 and OR 0.17 (0.1–0.6), *P* = .006, respectively; Table S10]. In multivariate analysis, both RSE > 108 grayscale value and FCP > 1520 µg/g remained significant predictors of endoscopic response, independent of prior exposure to more than one biologic or small molecule, male sex, and severe endoscopic disease (Table S11).

## 4. Discussion

In the current study, we found a significant association between submucosal hyper-echogenicity and histopathological presence of fat in UC patients. Subsequently, quantification of submucosal hyper-echogenicity by RSE was the only IUS parameter that predicted non-response before start of treatment in a prospective cohort of UC patients.

In Crohn’s disease (CD) chronic changes and fibrosis have been extensively studied and cross-sectional imaging techniques have most potential to quantify chronicity.^9^ For UC, transmural evaluation is underreported as it has long been considered a predominant mucosal disease. However, Gordon *et al.* demonstrated chronic changes in resection specimens extending the mucosa.^4^ Additionally, IUS studies demonstrated the submucosa as the most affected and most responsive wall layer.^6^^,^^7^

In the current study, we demonstrated high proportions of submucosal fat and collagen in resection specimens. Subsequently, we investigated the association between RSE and histopathological parameters. In linear regression, RSE was associated with both submucosal collagen and fat. However, in multivariate analysis, only the association with fat remained significant. Furthermore, no significant correlation was found between collagen and RSE. Interestingly, this is in contrast to previous findings from colectomy specimens of UC patients, where the presence of fat was not reported.^4^

Importantly, we identified a baseline RSE cut-off value of >108 grayscale value as a significant predictor of treatment non-response. In contrast, current IUS parameters that predict treatment response rely on changes over time.^6^ Therefore, RSE could serve as a novel biomarker predicting treatment non-response as early as baseline. These findings can contribute to managing patients’ expectations when medical treatment is initiated.

In CD, fat and collagen deposition has been associated with chronic fibrotic changes and stenotic complications.^10–15^ At IUS, preservation of wall layer stratification and absence of CDS were positively associated with fibrotic changes.^16–18^ In our UC cohort, preservation of wall layer stratification was associated with fat and fibrotic changes and is in line with these previous findings. Although promising, previous studies also demonstrated a poor to moderate reproducibility of wall layer stratification.^19^^,^^20^ Furthermore, submucosal hyper-echogenicity has also been observed in CD, but was difficult to score semi-quantitatively.^21^ With the RSE, we created a continuous parameter that is independent of machine settings, gain, and depth. Furthermore, inter-observer agreement was substantially higher for RSE than has been demonstrated for wall layer stratification.^19^^,^^20^ However, potential inter-machine variability remains unknown and requires further validation across different ultrasound systems.

The exact pathophysiology of submucosal fat deposition is not yet known in UC.^3^^,^^11^^,^^14^^,^^22^^,^^23^ In our study, no differences in fat deposition were observed between patients with inflammatory disease (UC and diverticulitis) and those without (cancer and refractory obstipation). However, RSE was higher in patients who failed more than one biological compared with those who had failed one or none, in both cohorts. This supports the role of a chronic, uncontrolled inflammation as a cause of fat deposition, rather than factors such as aging or disease duration. Consistently, age and disease duration did not influence submucosal fat deposition, which is also in line with previous research.^3^^,^^24^ It remains unclear whether the accumulating fat, if caused by inflammation, is a direct consequence of inflammation or rather a protective mechanism aimed at shielding the mucosa or deeper layers of the bowel wall from inflammation. Alternatively, fat deposition might inhibit the effectiveness of treatment and contribute to ongoing inflammation by preventing medication from adequately penetrating into the inflamed mucosa.

Interestingly, male sex was significantly associated with a higher RSE in Cohort 2, and in Cohort 1 male subjects scored significantly higher on submucosal presence of fat. The effect of sex on the disease course in IBD remains unclear, and does not explain our similar findings in non-IBD patients.^15^ In general, men tend to accumulate more abdominal visceral fat compared with pre-menopausal women, which may contribute to intramural fat deposition.^25^ Moreover, increased intra-abdominal visceral adipose tissue (as a percentage of total body mass) was reported to be independently associated with worse IBD outcomes, including lower rates of corticosteroid-free deep remission and endoscopic remission.^26^ Although associations between abdominal obesity and submucosal fat accumulation have been reported, we did not find a relationship between RSE and BMI.^23^ However, BMI may not be a reliable marker of visceral fat. For example, one study found visceral adiposity to be associated with decreased time to IBD flare, where BMI was not.^27^ Future translational studies are needed to further understand the mechanisms behind submucosal fat deposition.

Our study had some limitations. First, the samples sizes in the separate cohorts were relatively small, reducing statistical power and limiting the feasibility of sub-group analyses. Nevertheless, we selected the patient cohorts to represent both negative (cancer) as well as positive controls (diverticulitis) of chronic inflammation and we included a total of 118 patients. Second, we used a non-validated semi-quantitative method for scoring most of the histopathological parameters, which may have introduced bias in our primary outcome. However, no validated scoring system exists to quantify fat and collagen deposition and the scoring was performed by two independent, blinded pathologists. While inter-observer agreement was moderate for submucosal fat and fair to moderate for other features, repeating the primary analysis using each pathologist’s scores separately showed consistent results. Third, while the use of a single ultrasound system with one standardized software at one center ensured consistency and homogeneity of the results, it may limit generalizability, and cross-system reproducibility of RSE is still needed.

This study had also several strengths: we included both negative and positive control groups to establish our histopathology findings, we incorporated a second reader for both IUS and histopathology in Cohort 1 with corresponding inter-rater agreements, and we validated RSE in a separate prospectively collected cohort.

In conclusion, hyper-echogenicity of the submucosa on IUS in UC results from fat deposition. RSE provides a quantitative measure of submucosal echogenicity and serves as a reliable predictor of treatment non-response. These findings can contribute to better management of UC patients.

## Supplementary Material

jjaf158_Supplementary_Data

## Data Availability

Data, analytic methods, and study materials will be made available to other researchers upon reasonable request.
