# Supplementary material for: Submucosal hyper-echogenicity on intestinal ultrasound is associated with fat deposition and predicts treatment non-response in patients with ulcerative colitis
Source: J Crohns Colitis. 2025 Nov 4;19(10):jjaf158. doi: 10.1093/ecco-jcc/jjaf158 (PMC12596728; doi:10.1093/ecco-jcc/jjaf158)

Supplementary figure 1 – Measurements of relative submucosal layer echogenicity, grayscale values (0-255) in Radiant DICOM viewer


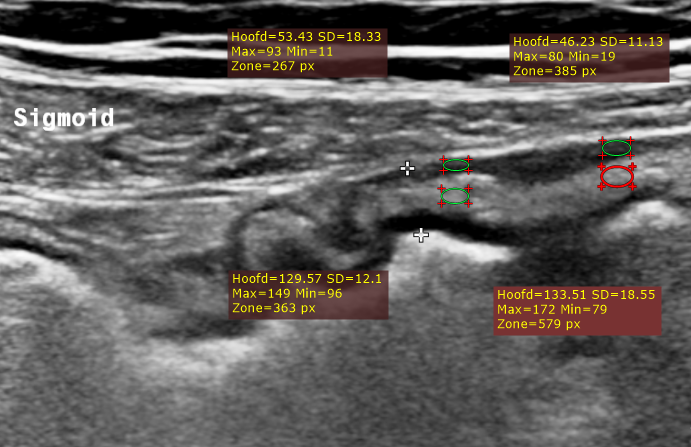


Muscularis propria

Submucosa

Mucosa

Lumen

**Sigmoid**


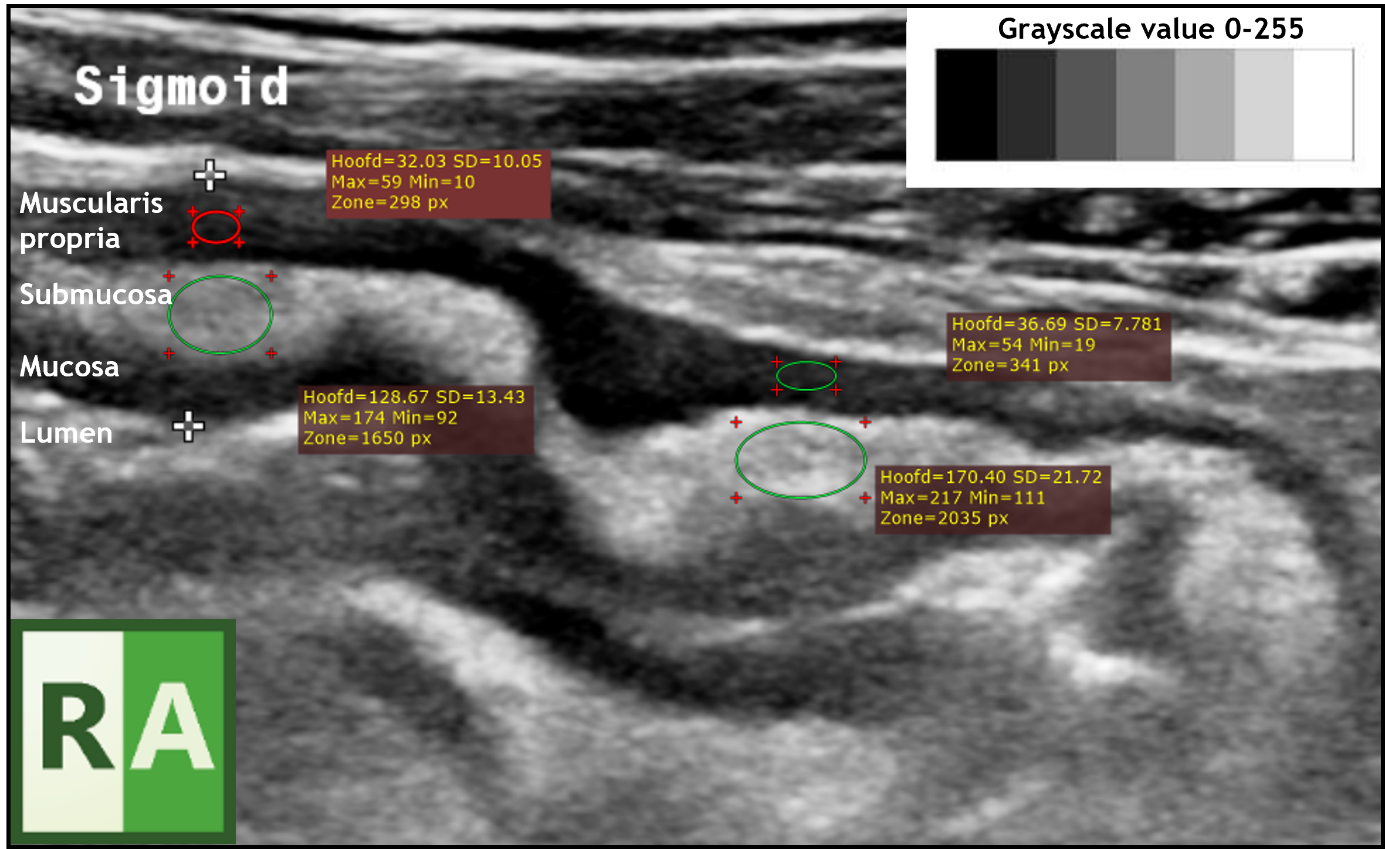

Supplement: jjaf158_Supplementary_Data [file jjaf158_supplementary_data.zip › Supplementary Figure 1.docx]
