# Supplementary material for: Submucosal hyper-echogenicity on intestinal ultrasound is associated with fat deposition and predicts treatment non-response in patients with ulcerative colitis
Source: J Crohns Colitis. 2025 Nov 4;19(10):jjaf158. doi: 10.1093/ecco-jcc/jjaf158 (PMC12596728; doi:10.1093/ecco-jcc/jjaf158)
Supplement: jjaf158_Supplementary_Data [file jjaf158_supplementary_data.zip › Supplementary Figure 2.docx]

Total inclusions

(n=118)

Colectomy patients

Cohort 1

(n=71)

Prospective study

Cohort 2

(n=47)

UC patients (n=19)

Non-IBD - matched with UC patients for age and sex (n=18)

Non-IBD - age >60 years (n=19)

Non-IBD **–** Diverticulitis (n=17)

Supplementary figure 2 – patient cohorts [UC: Ulcerative Colitis; IBD: Inflammatory Bowel Diseases]
