# Supplementary material for: Submucosal hyper-echogenicity on intestinal ultrasound is associated with fat deposition and predicts treatment non-response in patients with ulcerative colitis
Source: J Crohns Colitis. 2025 Nov 4;19(10):jjaf158. doi: 10.1093/ecco-jcc/jjaf158 (PMC12596728; doi:10.1093/ecco-jcc/jjaf158)
Supplement: jjaf158_Supplementary_Data [file jjaf158_supplementary_data.zip › Supplementary Figure 4.docx]

Supplementary Figure 4 – Differences in median baseline RSE in patients failing up to 3 biologics prior to treatment [RSE: relative submucosal echogenicity]
