# Supplementary material for: Submucosal hyper-echogenicity on intestinal ultrasound is associated with fat deposition and predicts treatment non-response in patients with ulcerative colitis
Source: J Crohns Colitis. 2025 Nov 4;19(10):jjaf158. doi: 10.1093/ecco-jcc/jjaf158 (PMC12596728; doi:10.1093/ecco-jcc/jjaf158)
Supplement: jjaf158_Supplementary_Data [file jjaf158_supplementary_data.zip › Supplementary Table 1.docx]

| **IUS parameter** | **Measurement** |
| --- | --- |
| Bowel wall thickness (BWT) | 4 measurements from lumen-mucosa interface up to muscularis propria-serosa interface (2× measurement longitudinal (≥10 mm between measurements) + 2× measurement cross-sectional plane (≥90° between measurements))/4 |
| Colour Doppler signal (CDS) | Categories  0: no Doppler signal (mLimberg 0)  1: 1 or 2 single vessels (mLimberg 1)  2: >2 single vessels or stretches limited to the wall (mLimberg 2)  3: stretches extending into the mesentery (mLimberg 3) |
| Haustrations | Categories  1: preserved haustrations  2: loss of haustrations |
| Wall layer stratification (WLS) | Categories  1: extensive loss of wall layer stratification (>3 cm)  2: focal loss of wall layer stratification (≤3 cm)  3: preserved wall layer stratification  4: hyperechoic wall layer stratification |
| Relative submucosal echogenicity (RSE) | Difference in grayscale values (0-255) in longitudinal mean areal grayscale values measurements between the submucosa and muscularis propria in a perpendicular fashion (RSE = mean areal grayscale values submucosa – mean areal grayscale values muscularis propria) |
| Fatty wrapping | Categories  1: absence of fatty wrapping  2: presence of fatty wrapping |
| Mesenteric lymph nodes | Categories  1: absence of lymph nodes  2: presence of lymph nodes ≤5.0 mm in the shortest axis  3: presence of lymph nodes >5.0 mm in the shortest axis |

Supplementary table 1 – IUS parameters [BWT: Bowel wall thickness; CDS: Colour Doppler Signal; mLimberg: modified Limberg score; WLS: Wall layer stratification; RSE: Relative submucosal echogenicity]
