# Supplementary material for: Submucosal hyper-echogenicity on intestinal ultrasound is associated with fat deposition and predicts treatment non-response in patients with ulcerative colitis
Source: J Crohns Colitis. 2025 Nov 4;19(10):jjaf158. doi: 10.1093/ecco-jcc/jjaf158 (PMC12596728; doi:10.1093/ecco-jcc/jjaf158)
Supplement: jjaf158_Supplementary_Data [file jjaf158_supplementary_data.zip › Supplementary Table 10.docx]

| Logistic regression for parameters associated with a RSE >108 grayscale value | | | | |
| --- | --- | --- | --- | --- |
| Variables at baseline | Univariable Analysis  OR (95% CI) | p-value | Multivariable Analysis  OR (95% CI) | p-value |
| Age | 1.01 (0.97-1.05) | 0.626 | - | - |
| Disease duration | 1.02 (0.95-1.09) | 0.671 | - | - |
| Sex (male vs female) | 4.74 (1.37-16.45) | **0.014** | 6.36 (1.37-29.54) | **0.018** |
| Failed >1 biological | 4.33 (1.21-15.44) | **0.024** | 4.13 (0.88-19.28) | 0.071 |
| BMI (kg/m^2^) | 0.95 (0.83-1.09) | 0.953 | - | - |
| SCCAI | 0.87 (0.72-1.03) | 0.108 | - | - |
| Active clinical disease (SCCAI ≥ 5) | 0.18 (0.02-1.69) | 0.134 | - | - |
| Severe endoscopic disease (EMS = 3) | 0.17 (0.05-0.60) | **0.006** | 0.21 (0.05-0.91) | **0.037** |

Supplementary Table 11 – Logistic regression for association between patient baseline characteristics and a RSE of >108 grayscale value. Backward selection.
