# Supplementary material for: Submucosal hyper-echogenicity on intestinal ultrasound is associated with fat deposition and predicts treatment non-response in patients with ulcerative colitis
Source: J Crohns Colitis. 2025 Nov 4;19(10):jjaf158. doi: 10.1093/ecco-jcc/jjaf158 (PMC12596728; doi:10.1093/ecco-jcc/jjaf158)
Supplement: jjaf158_Supplementary_Data [file jjaf158_supplementary_data.zip › Supplementary Table 11.docx]

| Logistic regression for endoscopic response (EMS decrease of 1 or more) | | | | |
| --- | --- | --- | --- | --- |
| Variables at baseline | Univariable Analysis  OR (95% CI) | p-value | Multivariable Analysis  OR (95% CI) | p-value |
| RSE >108 (grayscale values) | 0.119 (0.027-0.516) | **0.004** | 0.071 (0.008-0.662) | **0.020** |
| FCP >1520 (µg/g) | 3.750 (1.011-13.91) | **0.048** | 7.229 (1.089-47.972) | **0.041** |
| Sex (male vs female) | 0.975 (0.291-3.262) | 0.967 | 2.147 (0.381-12.109) | 0.387 |
| Failed >1 biological | 0.494 (0.145-1.684) | 0.260 | 1.115 (0.193-6.442) | 0.903 |
| Severe endoscopic disease (EMS = 3) | 2.083 (0.588-7.383) | 0.256 | 1.720 (0.259-11.435) | 0.575 |

Supplementary Table 11 – Results of logistic regression analysis for endoscopic response (EMS decrease of 1 or more) of clinical, biochemical and IUS parameters at baseline.
