# Supplementary material for: Submucosal hyper-echogenicity on intestinal ultrasound is associated with fat deposition and predicts treatment non-response in patients with ulcerative colitis
Source: J Crohns Colitis. 2025 Nov 4;19(10):jjaf158. doi: 10.1093/ecco-jcc/jjaf158 (PMC12596728; doi:10.1093/ecco-jcc/jjaf158)
Supplement: jjaf158_Supplementary_Data [file jjaf158_supplementary_data.zip › Supplementary Table 2.docx]

| **Histopathological parameter** | **Measurement** |
| --- | --- |
| Mucosal inflammation | Categories (Nancy score)  0: no or mild increase in chronic inflammatory cells number  1: presence of an increase in chronic inflammatory cells number that are easily apparent  2: few or rare neutrophils in lamina propria or in the epithelium that are difficult to see  3: presence of multiple clusters of neutrophils in lamina propria and/or in epithelium that are easily apparent  4: presence of mucosal ulceration defined by the loss of colonic crypts replaced with ‘immature’ granulation tissue (defined as disorganised blood vessels with extravasated neutrophils) or the presence of fibrinopurulent exudate |
| Submucosal inflammation | Categories  0: comparable to normal colonic submucosa.  1: at least one neutrophil was seen in the submucosa  2: more than one neutrophil was present in one High Power Field (HPF)  3: more than one HPF showed inflammation or groups of neutrophils were present |
| Submucosal fat | Categories  0: comparable to normal colonic submucosa.  1: more than one fat cell present per HPF  2: up to 50% of the submucosa is replaced by fat cells  3: >50% of the submucosa is replaced by fat cells |
| Submucosal collagen | Categories  0: comparable to normal colonic submucosa.  1: presence of thin wavy collagen fibers  2: presence of thick straight collagen fibers  3: >50% of the submucosa was filled by thick straight collagen fibers |

Supplementary table 2 – Histopathological parameters [HPF: High Power Field]
