# Supplementary material for: Submucosal hyper-echogenicity on intestinal ultrasound is associated with fat deposition and predicts treatment non-response in patients with ulcerative colitis
Source: J Crohns Colitis. 2025 Nov 4;19(10):jjaf158. doi: 10.1093/ecco-jcc/jjaf158 (PMC12596728; doi:10.1093/ecco-jcc/jjaf158)
Supplement: jjaf158_Supplementary_Data [file jjaf158_supplementary_data.zip › Supplementary Table 3.docx]

| **Histopathological results in UC patients** | | | | | | | | | |
| --- | --- | --- | --- | --- | --- | --- | --- | --- | --- |
| **a) Submucosal fat deposition** | **No fat** | | **Slight** | | **Moderate** | | **Significant** | | **All** |
| Extensive loss of WLS | 6 | | 0 | | 0 | | 0 | | **6** |
| Focal loss of WLS | 1 | | 0 | | 0 | | 0 | | **1** |
| Normal WLS | 0 | | 1 | | 0 | | 1 | | **2** |
| Hyperechoic WLS | 0 | | 5 | | 2 | | 3 | | **10** |
| **Total** | **7** | | **6** | | **2** | | **4** | | **19** |
|  | | | | | | | | | |
| **b) Submucosal collagen** | **No collagen** | | **Slight** | | **Moderate** | | **Significant** | | **All** |
| Extensive loss of WLS | 1 | | 3 | | 2 | | 0 | | **6** |
| Focal loss of WLS | 0 | | 0 | | 1 | | 0 | | **1** |
| Normal WLS | 0 | | 1 | | 0 | | 1 | | **2** |
| Hyperechoic WLS | 0 | | 1 | | 6 | | 3 | | **10** |
| **Total** | **1** | | **5** | | **9** | | **4** | | **19** |
|  | | | | | | | | | |
| **c) Submucosal inflammation** | **No inflammation** | | **Slight** | | **Moderate** | | **Significant** | | **All** |
| Extensive loss of WLS | 0 | | 1 | | 2 | | 3 | | **6** |
| Focal loss of WLS | 0 | | 0 | | 1 | | 0 | | **1** |
| Normal WLS | 1 | | 0 | | 0 | | 1 | | **2** |
| Hyperechoic WLS | 0 | | 3 | | 6 | | 1 | | **10** |
| **Total** | **1** | | **4** | | **9** | | **5** | | **19** |
|  | | | | | | | | | |
| **d) Nancy score** | **Nancy 0** | **Nancy 1** | | **Nancy 2** | | **Nancy 3** | | **Nancy 4** | **All** |
| Extensive loss of WLS | 0 | 0 | | 0 | | 0 | | 6 | **6** |
| Focal loss of WLS | 0 | 0 | | 0 | | 0 | | 1 | **1** |
| Normal WLS | 0 | 1 | | 0 | | 1 | | 0 | **2** |
| Hyperechoic WLS | 0 | 1 | | 0 | | 4 | | 5 | **10** |
| **Total** | **0** | **2** | | **0** | | **5** | | **12** | **19** |

Supplementary table 3a-d – Histopathological parameters compared to WLS [UC: Ulcerative Colitis; WLS: wall layer stratification]
