# Supplementary material for: Submucosal hyper-echogenicity on intestinal ultrasound is associated with fat deposition and predicts treatment non-response in patients with ulcerative colitis
Source: J Crohns Colitis. 2025 Nov 4;19(10):jjaf158. doi: 10.1093/ecco-jcc/jjaf158 (PMC12596728; doi:10.1093/ecco-jcc/jjaf158)
Supplement: jjaf158_Supplementary_Data [file jjaf158_supplementary_data.zip › Supplementary Table 4.docx]

|  | **Linear regression analysis for relative submucosal echogenicity**  **(grayscale value)** | | | | | | | | | | | |
| --- | --- | --- | --- | --- | --- | --- | --- | --- | --- | --- | --- | --- |
| **Variable** | Univariable | | | | | | Multivariable | | | | | |
|  | B | 95% CI | SE | Beta | t | p-value | B | 95% CI | SE | Beta | t | p-value |
| Submucosal fat | 30.38 | 17.28-43.48 | 6.21 | 0.77 | 4.89 | **<0.001** | 31.27 | 16.56-45.98 | 6.90 | 0.79 | 4.53 | **<0.001** |
| Submucosal collagen | 27.48 | 2.75-52.2 | 11.72 | 0.49 | 2.34 | **0.031** | 14.89 | -3.05-32.84 | 8.42 | 0.27 | 1.77 | 0.097 |
| Submucosal inflammation | -10.99 | -38.40-16.41 | 12.99 | -0.20 | -0.85 | 0.409 | 11.69 | -7.62-30.99 | 9.06 | 0.21 | 1.29 | 0.217 |

Supplementary Table 4 – Results of linear regression analysis showing associations between relative submucosal echogenicity (RSE; grayscale value) and histological features of the submucosal (fat, collagen and inflammation).
