# Supplementary material for: Submucosal hyper-echogenicity on intestinal ultrasound is associated with fat deposition and predicts treatment non-response in patients with ulcerative colitis
Source: J Crohns Colitis. 2025 Nov 4;19(10):jjaf158. doi: 10.1093/ecco-jcc/jjaf158 (PMC12596728; doi:10.1093/ecco-jcc/jjaf158)
Supplement: jjaf158_Supplementary_Data [file jjaf158_supplementary_data.zip › Supplementary Table 5.docx]

|  | **UC colectomy group** | | |  | **Control group 1**  **non-IBD matched** | | |  | **Control group 2 –**  **non-IBD elderly** | | |  | **Control group 3 –**  **diverticulitis** | | |
| --- | --- | --- | --- | --- | --- | --- | --- | --- | --- | --- | --- | --- | --- | --- | --- |
|  | Inflammation | Fat | Collagen |  | Inflammation | Fat | Collagen |  | Inflammation | Fat | Collagen |  | Inflammation | Fat | Collagen |
| Score 0 = none | 1 | 7 | 1 |  | 18 | 5 | 0 |  | 17 | 5 | 0 |  | 15 | 3 | 1 |
| Score 1 = slight | 4 | 6 | 5 |  | 0 | 3 | 4 |  | 0 | 4 | 1 |  | 0 | 5 | 5 |
| Score 2 = moderate | 9 | 2 | 9 |  | 0 | 8 | 14 |  | 0 | 6 | 13 |  | 1 | 6 | 10 |
| Score 3 = significant | 5 | 4 | 4 |  | 0 | 2 | 0 |  | 0 | 2 | 3 |  | 1 | 3 | 1 |
| **Total** | **19** | **19** | **19** |  | **18** | **18** | **18** |  | **17** | **17** | **17** |  | **17** | **17** | **17** |

Supplementary Table 5 – Histopathological findings of the submucosa (inflammation, fat and collagen) in all colectomy patients.
