# Supplementary material for: Submucosal hyper-echogenicity on intestinal ultrasound is associated with fat deposition and predicts treatment non-response in patients with ulcerative colitis
Source: J Crohns Colitis. 2025 Nov 4;19(10):jjaf158. doi: 10.1093/ecco-jcc/jjaf158 (PMC12596728; doi:10.1093/ecco-jcc/jjaf158)
Supplement: jjaf158_Supplementary_Data [file jjaf158_supplementary_data.zip › Supplementary Table 6.docx]

|  | UC patients | Non-IBD - age and sex matched | Non-IBD - elderly | Non-IBD - Diverticulitis | *p-value* |
| --- | --- | --- | --- | --- | --- |
| Number of patients | 19 | 18 | 17 | 17 | - |
| Age in years (median, IQR) | 39 (26-48) | 41 (26-51) | 78 (70-85) | 69 (60-76) | 0.704^a^ |
| Female sex | 8 (42%) | 9 (50%) | 8 (47%) | 9 (53%) | 0.926 |
| Submucosal fat | 12 (63%) | 13 (72%) | 12 (71%) | 14 (82%) | 0.648 |
| Submucosal collagen | 18 (95%) | 18 (100%) | 17 (100%) | 16 (94% | 0.570 |
| Submucosal Inflammation | 18 (95%) | 0 (0%) | 0 (0%) | 2 (12%) | **<0.001** |

Supplementary Table 6 – Comparison of the characteristics of Cohort 1 [UC: ulcerative colitis; IBD: inflammatory bowel diseases; IQR: inter-quartile range]

^a^p-value for Mann-Whitney U test between UC patients and age matched group
