# Supplementary material for: Submucosal hyper-echogenicity on intestinal ultrasound is associated with fat deposition and predicts treatment non-response in patients with ulcerative colitis
Source: J Crohns Colitis. 2025 Nov 4;19(10):jjaf158. doi: 10.1093/ecco-jcc/jjaf158 (PMC12596728; doi:10.1093/ecco-jcc/jjaf158)
Supplement: jjaf158_Supplementary_Data [file jjaf158_supplementary_data.zip › Supplementary Table 7.docx]

|  | No fat (n=20) | Slight fat (n=18) | Moderate fat (n=22) | Significant fat (n=11) | p-value |
| --- | --- | --- | --- | --- | --- |
| Age at time of colectomy | 55 (36-79) | 64 (39-76) | 60 (42-73) | 49 (26-62) | 0.083^a^ |
| Sex female | 12 (60%) | 12 (67%) | 6 (27%) | 4 (36%) | **0.045** |
| Inflammatory disease   - UC + diverticulitis - Non-inflammatory | 10  10 | 11  7 | 8  14 | 7  4 | 0.323 |

Supplementary Table 7 – Characteristics of patients with fat scores 0-3 in all Cohort 1 patients (n=71).^a^Lowest p-value for Mann-Whitney U test, between group 1 vs. 3 (slight fat vs. significant fat)
