# Supplementary material for: Submucosal hyper-echogenicity on intestinal ultrasound is associated with fat deposition and predicts treatment non-response in patients with ulcerative colitis
Source: J Crohns Colitis. 2025 Nov 4;19(10):jjaf158. doi: 10.1093/ecco-jcc/jjaf158 (PMC12596728; doi:10.1093/ecco-jcc/jjaf158)
Supplement: jjaf158_Supplementary_Data [file jjaf158_supplementary_data.zip › Supplementary Table 8.docx]

| Inter-rater agreement | Agreement  (95% CI) | p-value |
| --- | --- | --- |
| Relative submucosal echogenicity (RSE) | ICC = 0.81 (0.56-0.92) | **<0.001** |
| Nancy histopathology index (0-4) | κ =0.41 (0.05-0.77) | **0.012** |
| Submucosal inflammation (0-3) | κ =0.22 (-0.2-0.47) | **0.031** |
| Submucosal collagen (0-3) | κ =0.42 (0.17-0.67) | **0.005** |
| Submucosal fat (0-3) | κ =0.50 (0.30-0.71) | **<0.001** |

Supplementary Table 8 – Inter-rater agreement for RSE and histopathology
