# Supplementary material for: Submucosal hyper-echogenicity on intestinal ultrasound is associated with fat deposition and predicts treatment non-response in patients with ulcerative colitis
Source: J Crohns Colitis. 2025 Nov 4;19(10):jjaf158. doi: 10.1093/ecco-jcc/jjaf158 (PMC12596728; doi:10.1093/ecco-jcc/jjaf158)
Supplement: jjaf158_Supplementary_Data [file jjaf158_supplementary_data.zip › Supplementary Table 9.docx]

| **Correlations RSE** | | | |
| --- | --- | --- | --- |
| **Demographic characteristics** | *ρ (95% CI)* | *p-value* |  |
| Age | 0.047 (-0.25-0.34) | 0.754 |  |
| Disease duration | 0.028 (-0.27-0.32) | 0.851 |  |
| BMI baseline | 0.075 (-0.25-0.38) | 0.651 |  |
| CRP baseline | -0.396 (-0.67, -0.02) | **0.034** |  |
| FCP baseline | -0.030 (0.85, -0.33) | 0.847 |  |
| SCCAI baseline | -0.259 (-0.51-0.034) | 0.083 |  |
| EMS sigmoid baseline | -0.284 (-0.54-0.02) | 0.059 |  |
| **IUS parameters** | *ρ (95% CI)* | *p-value* | |
| BWT (mm) | -0.31 (-0.55-0.02) | **0.036** | |
| Submucosal thickness (mm) | -0.25 (-0.50-0.05) | 0.079 | |
| Colour Doppler Signal (mLimberg) | -0.211 (-0.49-0.10) | 0.169 | |
| Loss of wall layer stratification | -0.42 (-0.63, -0.14) | **0.004** | |
| Loss of haustration | -0.12 (-0.40-0.19) | 0.433 | |
| Presence of lymph nodes | -0.17 (-0.45-0.13) | 0.246 | |
| Presence of fatty wrapping | -0.22 (-0.48-0.08) | 0.139 | |

Supplementary Table 9 – Correlations between RSE and demographic characteristics and IUS parameters [RSE: relative submucosal echogenicitiy; BMI: body mass index; SCCAI: simple clinical colitis activity index; EMS: endoscopic mayo score; IUS: intestinal ultrasound; BWT: bowel wall thickness]
